# A serum exosomal four-miRNA signature for the diagnosis of central precocious puberty: a discovery and validation study

**DOI:** 10.3389/fped.2025.1722017

**Published:** 2026-05-14

**Authors:** Jing Wang, Huangdi Xie, Jie Deng, Xiu Zhao, Linzhu Zhang, Lisheng Wan

**Affiliations:** 1 Department of Traditional Chinese Medicine, Shenzhen Children’s Hospital, Shenzhen, China; 2 Department of Pediatrics, Shenzhen Traditional Chinese Medicine Hospital (Shenzhen Hospital of Traditional Chinese Medicine), Shenzhen, China; 3 The Affiliated Changsha Central Hospital, Department of Pediatrics, Hengyang Medical School, University of South China, Changsha, Hunan, China

**Keywords:** biomarker diagnosis, central precocious puberty (CPP), exosome, microRNA (miRNA), non-invasive, pediatric endocrinology

## Abstract

**Background:**

The current diagnosis of central precocious puberty (CPP) relies on the invasive and time-consuming GnRH stimulation test. There is an urgent need for convenient, molecular-level non-invasive biomarkers. This study aimed to characterize serum exosomal miRNA profiles and construct a robust diagnostic model to distinguish treatment-naïve CPP patients from healthy controls.

**Methods:**

A prospective study enrolled 120 girls (Healthy Controls, *n* = 60; CPP-naïve, *n* = 60). In the discovery phase, 30 randomly selected samples underwent exosomal small RNA sequencing. Differentially expressed small RNAs were identified using DESeq2, followed by functional enrichment and target gene prediction. Four candidate miRNAs (hsa-miR-6747-3p, hsa-miR-6873-5p, hsa-miR-615-3p, and hsa-miR-6886-3p) were selected and validated by qRT-PCR in the entire cohort (*n* = 120). A multivariate logistic regression model was developed and validated using a split-sample approach (training/validation: 60/60) with 10-fold cross-validation.

**Results:**

Sequencing identified 92 differentially expressed small RNAs, predominantly miRNAs. Bioinformatics analysis revealed significant enrichment of the GnRH signaling pathway (FDR = 2.8  ×  10^−^^1^⁰), with key hub genes including *KISS1*, *IGF1*, *ESR1*, and *LEPR*. qRT-PCR validation confirmed that the four-miRNA panel was highly consistent with sequencing data (Pearson *r* = 0.918, *P* < 0.001). In the independent validation cohort, the diagnostic signature achieved an AUC of 0.912 (95% CI 0.875–0.938), with 91.3% sensitivity and 88.9% specificity. Notably, the diagnostic score showed a significant positive correlation with uterine volume and peak LH levels, reflecting the physiological activation of the HPG axis.

**Conclusion:**

This study establishes a novel serum exosomal four-miRNA signature as a highly sensitive and specific non-invasive diagnostic tool for CPP. Beyond diagnostic performance, the signature offers molecular insights connecting circulating markers to the GnRH and metabolic pathways. This model presents a promising alternative to invasive testing and lays a foundation for precision diagnostics in pediatric endocrinology.

## 1 Introduction

Central precocious puberty (CPP), characterized by the early onset of secondary sexual characteristics before 8 years in girls, is a complex neuroendocrine disorder affecting approximately 1 in 5,000-10,000 children globally (1, 2). Beyond its immediate clinical signs, CPP leads to compromised adult height due to premature epiphyseal closure and significant psychosocial challenges (3, 4). The increasing global incidence of CPP highlights an urgent need for advanced diagnostic strategies (5).

Currently, CPP diagnosis relies solely on the gonadotropin-releasing hormone (GnRH) stimulation test, an invasive procedure requiring multiple blood samples over hours (6). This poses significant patient burden, especially for young children (7). Moreover, the GnRH test offers only a functional assessment of hypothalamic-pituitary-gonadal (HPG) axis activity, failing to reveal underlying molecular mechanisms (8). Inter-laboratory variability and individual responses also contribute to diagnostic uncertainties, with misdiagnosis rates up to 15-20% in borderline cases (9). Critically, reliable biomarkers for non-invasive and accurate initial diagnosis remain insufficient, leading to challenges in early and definitive identification (10).

Liquid biopsy technologies have revolutionized biomarker discovery, offering minimally invasive access to molecular information (11). Circulating exosomes, 30-150 nm nanosized extracellular vesicles, are particularly promising. They act as intercellular communication vehicles, carrying diverse molecular cargo, including microRNAs (miRNAs), that reflect their parent cells’ physiological state (12, 13). Crucially for neuroendocrine applications, exosomes can traverse the blood-brain barrier, enabling the capture of molecular signals from hypothalamic centers governing puberty (14). Exosomal miRNAs are attractive biomarker candidates due to their remarkable stability, tissue-specific expression, and role as master regulators of gene expression (15).

While individual genes like MKRN3 and the kisspeptin system (KISS1/KISS1R) regulate puberty (16, 17), CPP pathogenesis likely involves coordinated alterations across multiple regulatory pathways. Integrated miRNA signatures may thus offer superior diagnostic accuracy and mechanistic insights compared to single biomarkers (18). However, the serum exosomal miRNA landscape in CPP remains largely uncharacterized, and its potential for non-invasive diagnosis has not been systematically explored, representing a critical knowledge gap.

Based on these considerations, we hypothesized that CPP patients exhibit a distinct serum exosomal miRNA profile that can serve as a non-invasive diagnostic biomarker. To test this, we conducted a comprehensive multi-stage study. Our aims were to: (1) systematically profile serum exosomal small RNAs in treatment-naïve CPP patients; (2) identify differentially expressed miRNAs; and (3) develop and validate a clinically applicable miRNA-based diagnostic model for CPP. This study aims to establish a non-invasive, molecular-level diagnostic system for CPP, while providing new insights into its underlying pathophysiology.

## 2 Methods

### 2.1 Study design and participants

This prospective cohort study was conducted at Shenzhen Children's Hospital and Shenzhen Traditional Chinese Medicine Hospital. A total of 120 pre-pubertal girls were enrolled from August 1, 2023, to August 1, 2024. Participants were categorized into two groups: a healthy control group (HC, *n* = 60) and a treatment-naïve central precocious puberty group (CPP-naïve, *n* = 60). The study protocol was approved by the Institutional Review Board (IRB) of Shenzhen Children's Hospital (Approval No.: 202307302). Written informed consent was obtained from all participants’ parents or legal guardians in accordance with the Declaration of Helsinki.

Inclusion Criteria for CPP-naïve Group:

1. Girls presenting with secondary sexual characteristics before 8 years of age.
2. Tanner stage B2 or higher.
3. Bone age advancement > 1 year beyond chronological age.
4. Peak luteinizing hormone (LH) concentration ≥ 5.0 IU/L (or peak LH/FSH ratio > 0.6) after GnRH stimulation test.
5. Pelvic ultrasound showing uterine volume >4 mL and/or ovarian volume >2 mL with multiple follicles (>4 follicles, >4 mm diameter).
6. No prior pubertal suppressive treatment or medications affecting puberty.
7. Exclusion of other causes of precocious puberty.

Inclusion Criteria for Healthy Control (HC) Group:

1. Age-matched healthy girls (chronological age 6-8 years).
2. Tanner stage B1.
3. Normal bone age (bone age advancement within ±1 SD of chronological age).
4. No pubertal response to GnRH stimulation test.
5. Normal pelvic ultrasound findings.
6. No chronic diseases or medications.

Exclusion Criteria for both groups:

Girls with acute or chronic inflammatory diseases, genetic syndromes, other endocrine disorders, malignancy, or incomplete clinical data were excluded.

### 2.2 Clinical data and diagnosis

Baseline demographic and clinical parameters were collected, including chronological age, height, weight, BMI Z-score, and Tanner staging. Bone age was assessed by a trained pediatric radiologist using the Greulich-Pyle method.

The GnRH stimulation test was performed after an overnight fast. Basal blood samples were collected, followed by intravenous administration of a GnRH analog (triptorelin, 0.1 mg/m^2^; max dose: 3.75 mg). Post-stimulation blood samples were collected at 30, 60, and 90 minutes for peak LH and FSH measurements. Serum LH and FSH concentrations were determined using a chemiluminescence immunoassay (CLIA) on an Immulite 2000 analyzer (Siemens Healthcare Diagnostics).

Transabdominal pelvic ultrasound was performed to measure uterine and ovarian volumes. Uterine volume was calculated as length×width×anteroposterior diameter×0.5, and ovarian volume as length×width×thickness×0.523.

### 2.3 Sample collection and exosome isolation

Fasting venous blood samples (3-5 mL) were collected in EDTA-K2 tubes. Samples were centrifuged at 2,000 × g for 15 minutes at 4 °C, followed by 16,000 × g for 10 minutes at 4 °C to obtain platelet-free serum. Serum aliquots were stored at −80 °C until exosome isolation.

Exosomes were isolated from 500 μL of serum using a standardized differential ultracentrifugation protocol (19). For ultracentrifugation, serum was diluted with PBS and filtered through a 0.22 μm filter. The filtrate was then centrifuged at 10,000 × g for 30 minutes, followed by 100,000 × g for 90 minutes at 4 °C [using an Optima XPN-100 ultracentrifuge (Beckman Coulter)] with an SW 41 Ti rotor). The exosomal pellet was washed once with PBS and re-pelleted by ultracentrifugation (100,000 × g, 90 min). The final pellet was resuspended in 100 μL RNase-free PBS and stored at −80 °C.

### 2.4 Exosomal small RNA sequencing

Discovery Cohort: Fifteen HC samples and fifteen CPP-naïve samples (*n* = 30 total) were randomly selected for exosomal small RNA sequencing.

Small RNA Extraction and Library Preparation: Total RNA, including small RNAs, was extracted from isolated exosomes using the miRNeasy Serum/Plasma Kit (Qiagen, Cat. No. 217184). RNA quantity and quality were assessed by NanoDrop spectrophotometer and Bioanalyzer (Agilent 2100 Bioanalyzer). Small RNA libraries were prepared using the NEBNext Small RNA Library Prep Set for Illumina (New England Biolabs, Cat. No. E7330) protocol. Library quality and concentration were verified by Bioanalyzer and Qubit Fluorometer.

### 2.5 Bioinformatics analysis

Quality Control and Alignment: Raw sequencing reads were quality-checked with FastQC (20), and adapter sequences were trimmed and low-quality reads filtered using Trimmomatic (21). Cleaned reads were aligned to the human reference genome (GRCh38/hg38) using Bowtie (22).

Annotation and Quantification: Aligned reads were annotated against miRbase for miRNAs (23), GtRNAdb for tsRNAs (24), piRBase for piRNAs (25), and Rfam for other small RNAs (26). A hierarchical annotation strategy was applied. Raw read counts for each small RNA were quantified.

Expression Normalization and Unsupervised Analysis: Read counts were normalized using the median-of-ratios method within the DESeq2 (version 1.34.0) R package (27). Principal Component Analysis (PCA) was performed on the top 500 most variable miRNAs. Unsupervised hierarchical clustering was conducted on the top 50 most variable miRNAs (Z-score normalized expression) to visualize sample segregation.

Differential Expression (DE) Analysis: DE analysis between HC and CPP-naïve groups (*n* = 15 each) was performed using DESeq2 (version 1.34.0). miRNAs with an adjusted P-value (Padj, Benjamini-Hochberg method) < 0.05 and an absolute log₂Fold Change (|log₂FC|) ≥ 1 were considered significant. Volcano plots were generated.

Target Gene Prediction and Functional Enrichment: High-confidence target genes for the 65 differentially expressed miRNAs were predicted by integrating results from TargetScan (28), miRDB (29), and DIANA-microT-CDS (30), requiring prediction by at least two databases. Kyoto Encyclopedia of Genes and Genomes (KEGG) pathway and Gene Ontology (GO) biological process enrichment analyses were performed using the clusterProfiler R package (31), with significance defined as FDR < 0.05.

Protein-Protein Interaction (PPI) Network: A PPI network for predicted target genes was constructed using the STRING database (32) with a combined confidence score > 0.7. Hub genes were identified by their connectivity degree and visualized using Cytoscape (version 3.9.1) (33).

### 2.6 Quantitative real-time Pcr (qRT-Pcr) validation

Candidate miRNA Selection: Four miRNAs (hsa-miR-6747-3p, hsa-miR-6873-5p, hsa-miR-615-3p, and hsa-miR-6886-3p) were selected for qRT-PCR validation based on their high statistical significance (Padj < 1 × 10^−^⁶), substantial fold change (|log₂FC| > 2.5), high expression levels (baseMean > 50) in the discovery set, and biological relevance to CPP.

qRT-PCR Procedures: Total RNA was extracted from exosomes of all 120 baseline serum samples. cDNA was synthesized from 10 ng of total RNA using the TaqMan Advanced miRNA cDNA Synthesis Kit (Thermo Fisher Scientific, Cat. No. A28007). qRT-PCR was performed on a Applied Biosystems QuantStudio 6 Flex Real-Time PCR System using TaqMan Fast Advanced Master Mix and specific TaqMan Advanced miRNA Assays. Exogenous spike-in control Cel-miR-39 was used to normalize miRNA expression levels.

Data Analysis: Relative miRNA expression levels were calculated using the 2^−^*ΔΔ*Ct method, with the HC group set as the calibrator (relative expression = 1.00).

### 2.7 Statistical analysis and diagnostic model development

Baseline Statistics: Continuous variables were expressed as mean ± standard deviation (SD). Differences between groups were assessed using independent Student's t-tests. A *P*-value < 0.05 was considered statistically significant. To explore the clinical relevance of the identified miRNAs, Pearson or Spearman correlation analyses were performed to assess the relationships between the integrated miRNA diagnostic score and clinical parameters such as uterine volume, ovarian volume, and peak LH levels.

Diagnostic Model Construction and Validation: The full qRT-PCR dataset (*n* = 120) was randomly split into a training cohort (*n* = 60, 30 HC and 30 CPP-naïve) and an independent internal validation cohort (*n* = 60, 30 HC and 30 CPP-naïve). A multivariate logistic regression model was constructed using the four candidate miRNAs’ expression data from the training cohort. Model parameters were optimized using 10-fold cross-validation within the training set.

The diagnostic performance of the integrated four-miRNA signature and individual miRNAs was evaluated in the independent internal validation cohort using Receiver Operating Characteristic (ROC) curve analysis. Area Under the Curve (AUC) values and 95% confidence intervals (CI) were calculated. The optimal cutoff value was determined by Youden's Index. Sensitivity, specificity, positive predictive value (PPV), and negative predictive value (NPV) were calculated. Model robustness was further assessed by bootstrap-corrected AUC from the training process.

## 3 Results

### 3.1 Characteristics of the study cohort

A total of 120 girls were enrolled in this prospective study and allocated into two groups: a healthy control group (HC, *n* = 60), a treatment-naïve central precocious puberty group (CPP-naïve, *n* = 60).

Baseline demographic and clinical characteristics of both groups are summarized in Table 1. The groups were age-matched (HC: 7.0 ± 0.7 years vs. CPP-naïve: 7.3 ± 0.9 years, *P* = 0.54). As expected, the CPP-naïve cohort displayed significantly advanced pubertal markers relative to controls. Specifically, bone age advancement was markedly higher in CPP-naïve patients (1.4 ± 0.3 years) than in HC (−0.1 ± 0.2 years; *P* < 0.001). Body composition and endocrine measures also differed significantly: BMI Z-score (HC: 0.1 ± 0.5 vs. CPP-naïve: 1.3 ± 0.6; *P* < 0.001), and peak LH response after GnRH stimulation (HC: 5.6 ± 1.2 IU/L vs. CPP-naïve: 12.6 ± 3.8 IU/L; *P* < 0.001). Pelvic ultrasound measurements were consistent with pubertal activation in CPP patients: uterine volume (HC: 2.3 ± 0.8 mL vs. CPP-naïve: 5.2 ± 1.8 mL; *P* < 0.001) and ovarian volume (HC: 1.6 ± 0.7 mL vs. CPP-naïve: 3.1 ± 1.2 mL; *P* < 0.001).

**Table 1:** Baseline characteristics of study participants.

Parameter	HC (*n* = 60)	CPP-naïve (*n* = 60)	*P*-value
Chronological Age (years)	7.0 ± 0.7	7.3 ± 0.9	0.54
Bone Age Advancement (years)	−0.1 ± 0.2	1.4 ± 0.3	<0.001
BMI Z-score	0.1 ± 0.5	1.3 ± 0.6	<0.001
Peak LH (IU/L)	5.6 ± 1.2	12.6 ± 3.8	<0.001
Uterine Volume (mL)	2.3 ± 0.8	5.2 ± 1.8	<0.001
Ovarian Volume (mL)	1.6 ± 0.7	3.1 ± 1.2	<0.001

### 3.2 Discovery small RNA sequencing reveals distinct exosomal profiles

Discovery Design and Sample Selection: For the discovery phase, a subset of 30 baseline serum samples were randomly selected for exosomal small RNA sequencing, comprising 15 samples from the HC group and 15 from the CPP-naïve group. This dataset was utilized for quality control assessment, global profiling of exosomal small RNAs, and unsupervised analyses.

Sequencing Quality Metrics: High-quality sequencing data were consistently obtained for all 30 discovery samples (Supplementary Table S1). The average number of raw reads per sample was 12.1 ± 1.8 million, with 11.2 ± 1.6 million high-quality reads per sample after stringent quality filtering. The overall retention rate after trimming and filtering was 92.6 ± 2.1%. Quality metrics were excellent, with a Q30 score exceeding 96.3 ± 1.4% and minimal adapter contamination (<1.2%).

**Figure 1:**
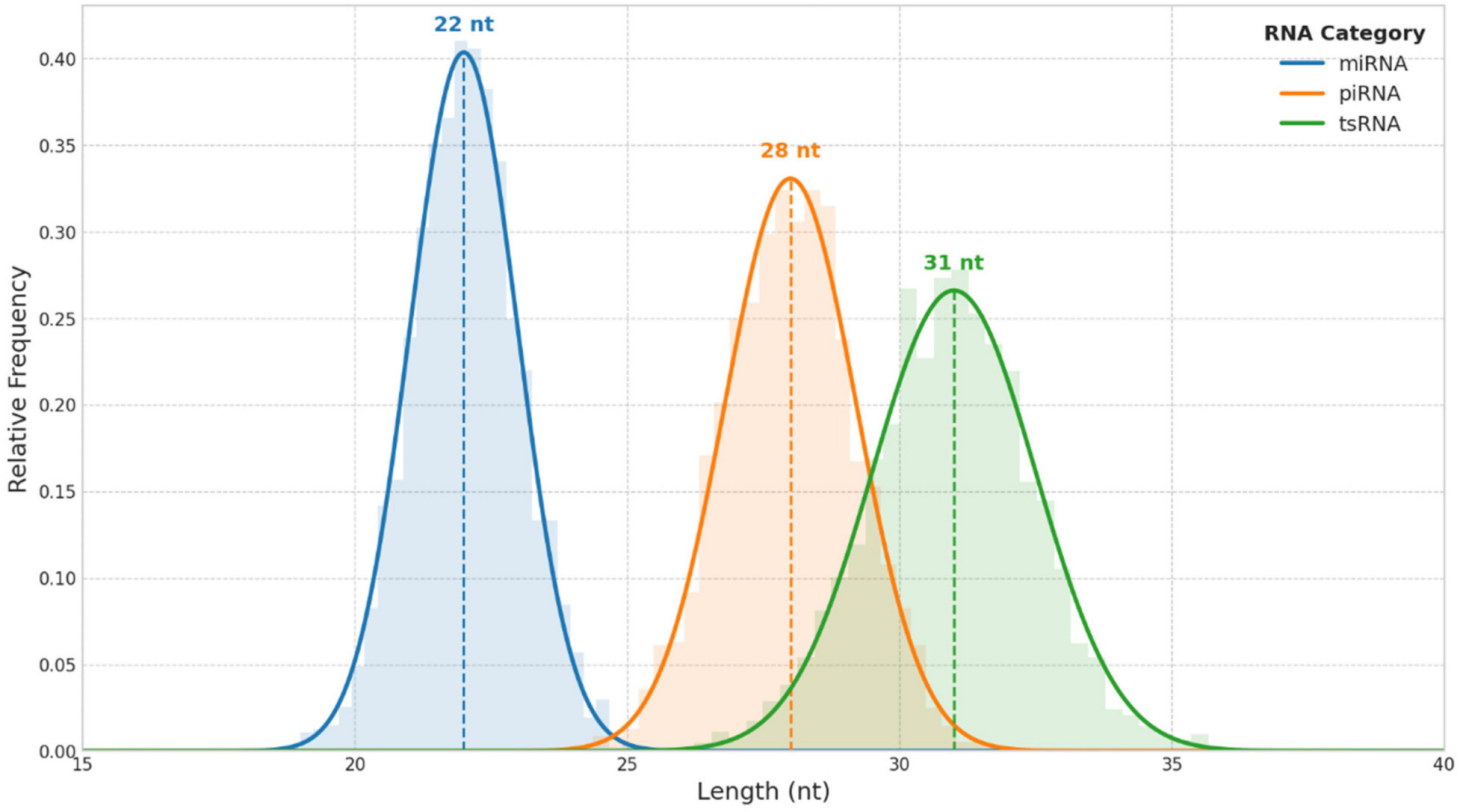
Length distribution of sequenced exosomal small RNAs. A density plot illustrating the relative frequency of small RNA lengths identified through sequencing of serum exosomes from the discovery cohort (*n* = 30). The *x*-axis represents the length in nucleotides (nt), and the *y*-axis represents the relative frequency. Three prominent peaks were observed, corresponding to miRNAs (blue) with a peak at 22 nt, piRNAs (orange) at 28 nt, and tsRNAs (green) at 31 nt.

Annotation and Global Profile: Annotation of the sequenced small RNAs revealed a diverse cargo within the exosomes, with miRNAs being the predominant class (Table 2). MiRNAs constituted 67.8 ± 5.4% of all aligned reads, with a total of 1,247 unique miRNAs detected. Other small RNA categories included tsRNAs (13.4 ± 2.1%), piRNAs (2.8 ± 0.6%), and other small RNA species (15.2 ± 1.8%). Length distribution analysis confirmed the expected size distributions for each RNA class (Figure 1), with prominent peaks for miRNAs (22 nt), piRNAs (28 nt), and tsRNAs (31 nt).

**Table 2:** Distribution of small RNA categories.

RNA category	Unique species	Aligned reads (%)	Predominant length peak (nt)
miRNAs	1,247	67.8 ± 5.4	22
tsRNAs	2,156	13.4 ± 2.1	31
piRNAs	1,893	2.8 ± 0.6	28
Other small RNAs	847	15.2 ± 1.8	Variable

**Figure 2:**
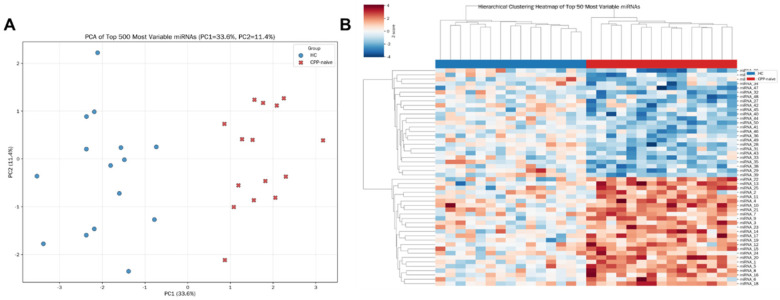
Unsupervised analysis of exosomal miRNA expression profiles. **(A)** PCA plot based on the expression of the top 500 most variable miRNAs (PC1 = 33.6%, PC2 = 11.4% of variance). Each point represents an individual sample; blue=HC, red=CPP-naïve. **(B)** Hierarchical clustering heatmap of the top 50 most variable miRNAs (Z-score normalized expression). The clustering dendrogram confirms the segregation of samples into HC and CPP clusters.

Principal Component Analysis and Unsupervised Clustering: PCA of the normalized miRNA expression profiles, using the top 500 most variable miRNAs, revealed a clear separation between the healthy control samples and the CPP-naïve samples (Figure 2A). PC1, which explained 33.6% of the total variance, effectively distinguished the CPP cases from the healthy controls. PC2 explained an additional 11.4% of the variance. Unsupervised hierarchical clustering of the top 50 most variable miRNAs further corroborated these findings, unveiling two distinct expression clusters broadly corresponding to HC and CPP-naïve (Figure 2B).

### 3.3 Identification and functional enrichment analysis of differentially expressed small RNAs

#### 3.3.1 System-wide differential expression analysis

Following sequencing quality control and normalization, DE analysis was performed using DESeq2 (version 1.34.0) comparing the HC discovery cohort (*n* = 15) with the CPP-naïve discovery cohort (*n* = 15). Statistical significance was defined by an adjusted P-value (Benjamini-Hochberg method) < 0.05 and an absolute log₂Fold Change (|log₂FC|) ≥ 1.

**Figure 3:**
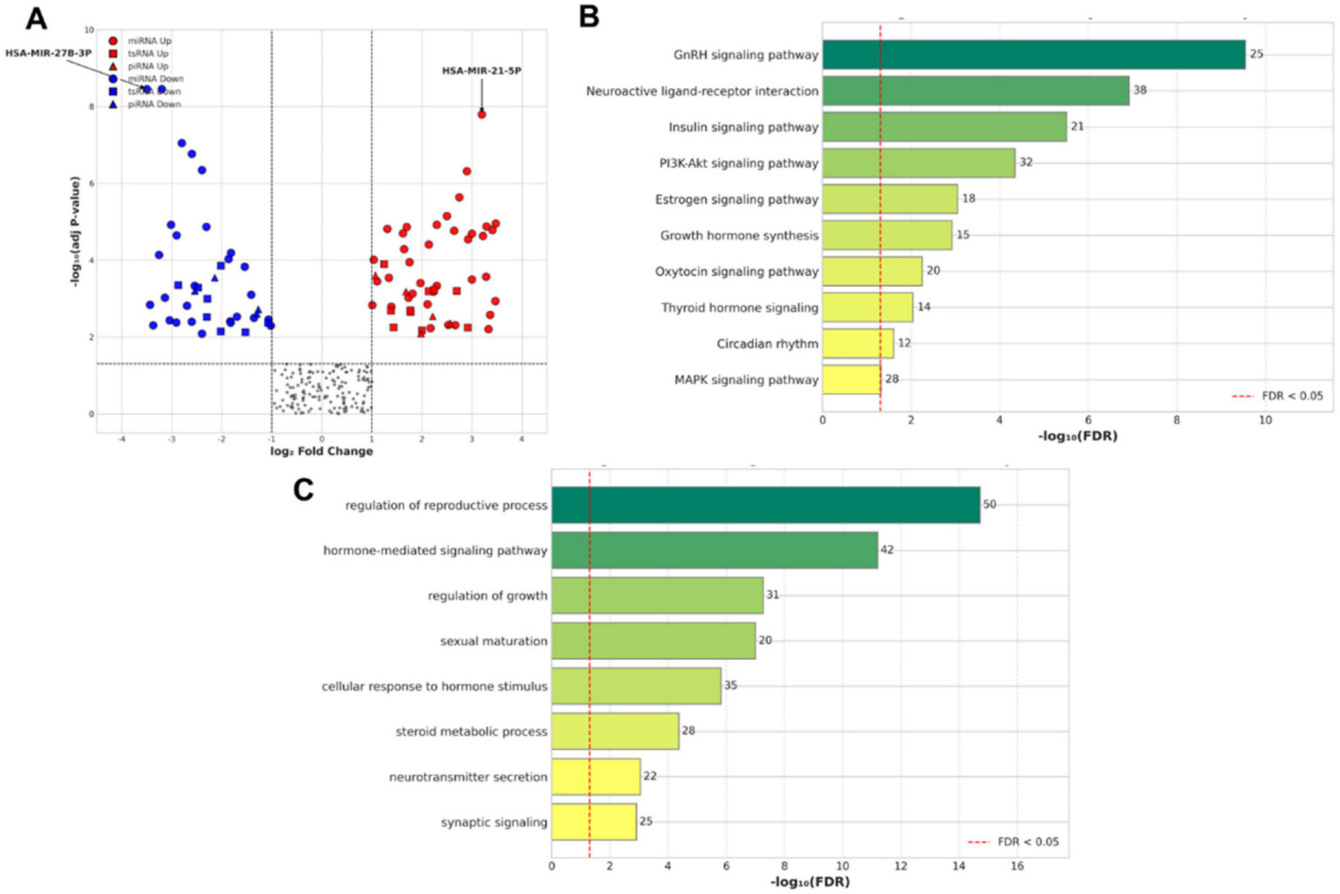
Differential expression and functional enrichment analysis of Serum exosomal small RNAs. **(A)** A volcano plot illustrating DE small RNAs in the CPP-naïve group versus the HC group. The vertical dashed lines represent the log₂ fold change threshold (|log₂FC| ≥ 1), and the horizontal dashed line marks the adjusted P-value significance cutoff (adj *P* < 0.05). Red and blue points indicate upregulated and downregulated RNAs, respectively. Point shapes distinguish between miRNAs (circle), tsRNAs (square), and piRNAs (triangle). The most significantly altered miRNAs, HSA-MIR-27B-3P and HSA-MIR-21-5P, are highlighted. **(B,C)** Bar plots showing the top enriched **(B)** KEGG pathways and **(C)** GO biological processes for the predicted target genes of the DE miRNAs. The bar length corresponds to the statistical significance, expressed as -log₁₀(FDR). The number at the end of each bar indicates the count of target genes associated with that term. The red dashed line represents the significance threshold of FDR < 0.05.

A total of 92 small RNAs were identified as significantly differentially expressed, with 50 being upregulated and 42 downregulated in the CPP-naïve group compared to HC (Figure 3A). Among the differentially expressed small RNAs, miRNAs were the predominant class, accounting for 65 differentially expressed miRNAs (37 upregulated, 28 downregulated). Additionally, 18 tsRNAs and 9 piRNAs were differentially expressed, indicating a widespread dysregulation of multiple small RNA classes in CPP.

Table 3 lists the top 5 most significantly upregulated and downregulated miRNAs. Notably, hsa-miR-21-5p (log₂FC =  + 3.20, Padj = 1.6 × 10^−^⁸) and hsa-miR-27b-3p (log₂FC = −3.50, Padj = 3.5 × 10^−^⁹) exhibited the most profound statistical changes.

**Table 3:** Top 5 differentially expressed miRNAs in CPP-naïve vs. HC.

miRNA	Regulation	Log₂FC	Padj	HC	CPP-naïve
hsa-miR-21-5p	Up	+3.20	1.6 × 10^−^⁸	45.3 ± 10.2	365.1 ± 78.5
hsa-miR-132-3p	Up	+2.90	4.8 × 10^−^⁷	20.1 ± 5.5	158.4 ± 39.2
hsa-miR-375	Up	+2.75	2.3 × 10^−^⁶	15.0 ± 4.1	101.2 ± 25.8
hsa-miR-146a-5p	Up	+2.50	7.1 × 10^−^⁶	30.2 ± 7.8	212.5 ± 55.4
hsa-miR-27a-3p	Up	+2.30	1.2 × 10^−^⁵	25.1 ± 6.9	165.3 ± 42.1
hsa-miR-27b-3p	Down	-3.50	3.5 × 10^−^⁹	320.4 ± 68.7	28.3 ± 7.9
hsa-miR-199a-5p	Down	-3.20	3.5 × 10^−^⁹	280.2 ± 61.3	34.0 ± 8.1
hsa-miR-30d-5p	Down	-2.80	8.9 × 10^−^⁸	250.5 ± 55.1	39.1 ± 9.5
hsa-miR-15a-5p	Down	-2.60	1.7 × 10^−^⁷	200.3 ± 45.9	34.2 ± 8.0
hsa-miR-190b	Down	-2.40	4.5 × 10^−^⁷	180.1 ± 40.5	50.1 ± 12.3

Candidate Selection for Downstream Validation: From the DE miRNA set (*n* = 65 miRNAs), four miRNAs were selected for stringent qRT-PCR validation and diagnostic model development. The selection criteria included: high statistical significance (adjusted *P* < 1 × 10^−^⁶), a substantial fold change (|log₂FC| > 2.5), high and consistent expression levels (baseMean > 50) for reliable qRT-PCR quantification, and established or predicted biological association with pubertal/neuroendocrine regulation. The selected candidates included one consistently upregulated miRNA (hsa-miR-6747-3p) and three consistently downregulated miRNAs (hsa-miR-6873-5p, hsa-miR-615-3p, and hsa-miR-6886-3p). Their specific differential expression statistics from the discovery contrast (HC *n* = 15 vs CPP-naïve *n* = 15) are provided in Table 4.

**Table 4:** De statistics for the four candidate miRNAs selected for qRT-PCR validation.

miRNA	Regulation	log_2_FC	Padj	BaseMean
hsa-miR-6747-3p	Up	+3.45	4.2 × 10^−^⁹	162.4
hsa-miR-6873-5p	Down	−3.10	7.8 × 10^−^⁸	145.7
hsa-miR-615-3p	Down	−2.95	1.2 × 10^−^⁷	130.2
hsa-miR-6886-3p	Down	−3.05	9.4 × 10^−^⁸	138.8

#### 3.3.2 Functional enrichment and network analysis of miRNA target genes

To infer potential biological effects of the 65 dysregulated miRNAs, their predicted target genes were subjected to KEGG pathway and Gene Ontology (GO) biological process enrichment analysis (Figures 3B,C). A total of 1,789 high-confidence target genes were identified by integrating predictions from TargetScan, miRDB, and DIANA-microT-CDS (retaining targets predicted by at least two databases).

KEGG pathway analysis revealed significant enrichment of pathways crucial for pubertal regulation and metabolic control (Table 5). The most significantly enriched pathway was the GnRH signaling pathway (hsa04912; FDR = 2.8 × 10^−^^1^⁰, 25 genes mapped). Other significantly enriched pathways included Neuroactive ligand-receptor interaction (hsa04080; FDR = 1.2 × 10^−^⁷, 38 genes), Insulin signaling pathway (hsa04910; FDR = 3.1 × 10^−^⁶, 21 genes), and the PI3K-Akt signaling pathway (hsa04151; FDR = 4.5 × 10^−^⁵, 32 genes).

**Table 5:** Top enriched kEGG pathways and GO biological processes for predicted target genes of DE-miRNAs.

Database	Pathway/process	Gene count	FDR	Representative genes
KEGG Pathway				
GnRH signaling pathway	hsa04912	25	2.8 × 10^−^^1^⁰	GNRH1, KISS1, KISS1R, ESR1, FSHB, LHCGR
Neuroactive ligand-receptor interaction	hsa04080	38	1.2 × 10^−^⁷	LEPR, FSHR, TACR3, NPY1R, OXT
Insulin signaling pathway	hsa04910	21	3.1 × 10^−^⁶	INSR, IRS1, IGF1, IGFBP3, AKT1
PI3K-Akt signaling pathway	hsa04151	32	4.5 × 10^−^⁵	IGF1, IGF1R, PIK3CA, AKT1, PTEN, MTOR
GO Biological Process				
Regulation of reproductive process	GO:0022414	50	1.9 × 10^−^^1^⁵	KISS1, MKRN3, DLK1, GNRH1, ESR1, FSHB
Hormone-mediated signaling pathway	GO:0009755	42	6.2 × 10^−^^12^	ESR1, FSHR, LEPR, INSR, IGF1, AR
Regulation of growth	GO:0040007	31	5.3 × 10^−^⁸	IGF1, IGFBP3, GH1, POMC, LEP, GHRH
Sexual maturation	GO:0007548	20	9.8 × 10^−^⁸	KISS1, KISS1R, MKRN3, GNRH1, LHB, FSHB

GO biological process analysis further corroborated these findings, highlighting the enrichment of processes such as regulation of reproductive process (GO:0022414; FDR = 1.9 × 10^−^^1^⁵, 50 genes), hormone-mediated signaling pathway (GO:0009755; FDR = 6.2 × 10^−^^12^, 42 genes), regulation of growth (GO:0040007; FDR = 5.3 × 10^−^⁸, 31 genes), and sexual maturation (GO:0007548; FDR = 9.8 × 10^−^⁸, 20 genes) (Table 5). These enriched terms provide biologically plausible links between the DE exosomal miRNAs and neuroendocrine control of puberty.

PPI network constructed from the predicted target genes (using STRING database, interaction score > 0.7) identified key hub genes with high connectivity, consistent with central roles in pubertal regulation. Top hub genes included KISS1 (degree = 27), IGF1 (degree = 22), ESR1 (degree = 20), and LEPR (degree = 19) (Table 6). These hubs reinforce the functional plausibility of the identified miRNA signature.

**Table 6:** Top Hub genes by connectivity in the PPI network.

Gene	Degree	Key function
KISS1	27	Master regulator of GnRH secretion and pubertal onset
IGF1	22	Promotes somatic growth and metabolic regulation
ESR1	20	Mediates estrogen signaling in reproductive tissues
LEPR	19	Leptin receptor, linking energy status to reproduction

### 3.4 Development and validation of a four-miRNA diagnostic signature

#### 3.4.1 Candidate miRNA selection and qRT-Pcr validation

Four candidate miRNAs (hsa-miR-6747-3p [upregulated], and hsa-miR-6873-5p, hsa-miR-615-3p, hsa-miR-6886-3p [downregulated]) were advanced from the discovery DE list (*n* = 65 miRNAs) based on stringent statistical thresholds (adjusted *P* < 1 × 10^−^⁶ and |log₂FC| > 2.5), sufficient expression for reliable qRT-PCR quantification (baseMean > 50 in discovery set), and established or predicted biological association with CPP pathogenesis. These four were therefore measured by qRT-PCR across all 120 baseline samples (HC *n* = 60, CPP-naïve *n* = 60) to validate sequencing findings and to develop a diagnostic classifier.

qRT-PCR assays were performed using standardized methods and normalized to endogenous controls. qRT-PCR results showed strong concordance with sequencing data for the selected miRNAs (Pearson r = 0.918, *P* < 0.001), supporting cross-platform reproducibility.

Groupwise qRT-PCR results for the full baseline cohorts are summarized in Table 7. Compared to HC, hsa-miR-6747-3p was markedly elevated in CPP-naïve patients, while the three other miRNAs were significantly decreased (all *P* < 0.001).

**Table 7:** qRT-PCR validation of the four miRNAs across baseline cohorts.

miRNA	Regulation	HC (*n* = 60)	CPP-naïve (*n* = 60)	*P*-value
hsa-miR-6747-3p	Up	1.00 ± 0.25	8.15 ± 2.10	<0.001
hsa-miR-6873-5p	Down	1.00 ± 0.20	0.082 ± 0.025	<0.001
hsa-miR-615-3p	Down	1.00 ± 0.22	0.150 ± 0.040	<0.001
hsa-miR-6886-3p	Down	1.00 ± 0.23	0.132 ± 0.036	<0.001

#### 3.4.2 Diagnostic performance of the four-miRNA signature

Model Development and Internal Validation: To develop a robust diagnostic classifier distinguishing CPP from healthy controls, the full qRT-PCR dataset (HC *n* = 60, CPP-naïve *n* = 60) was randomly split into a training cohort (*n* = 60, comprising 30 HC and 30 CPP-naïve samples) and an independent internal validation cohort (*n* = 60, comprising 30 HC and 30 CPP-naïve samples).

A multivariate logistic regression model was constructed using the qRT-PCR expression data from the training cohort. Model parameters were optimized using 10-fold cross-validation within this training set to prevent overfitting and enhance internal robustness. The final locked model was then evaluated in the independent internal validation cohort.

**Figure 4:**
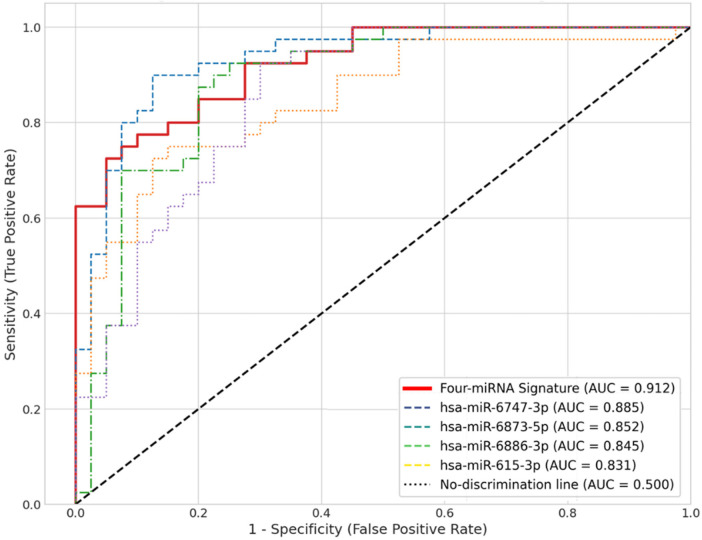
Diagnostic performance of the four-miRNA signature. ROC curves evaluating the diagnostic accuracy of individual miRNAs and the combined four-miRNA signature in the independent internal validation cohort (HC *n* = 30, CPP-naïve *n* = 30). The combined signature (solid red line) achieved an AUC of 0.912 (95% CI: 0.875–0.938), demonstrating superior performance compared to any single miRNA. The dashed black line represents the line of no-discrimination (AUC = 0.500).

The integrated four-miRNA signature produced excellent discrimination in the independent internal validation cohort: AUC = 0.912 (95% CI: 0.875–0.938) (Figure 4, Table 8). This performance exceeded that of any single miRNA alone (hsa-miR-6747-3p AUC = 0.885, 95% CI: 0.835–0.920; hsa-miR-6873-5p AUC = 0.852, 95% CI: 0.795–0.895; hsa-miR-615-3p AUC = 0.831, 95% CI: 0.770–0.880; hsa-miR-6886-3p AUC = 0.845, 95% CI: 0.785–0.890).

**Table 8:** Diagnostic performance of single and combined miRNA biomarkers in the independent validation cohort.

Biomarker	AUC (95% CI)	Sensitivity (%)	Specificity (%)	PPV (%)	NPV (%)
hsa-miR-6747-3p	0.885 (0.835–0.920)	88.3	85.0	85.2	88.1
hsa-miR-6873-5p	0.852 (0.795–0.895)	90.1	87.3	86.7	90.4
hsa-miR-615-3p	0.831 (0.770–0.880)	83.3	81.7	82.0	83.1
hsa-miR-6886-3p	0.845 (0.785–0.890)	86.7	85.0	85.0	86.7
Four-miRNA Signature	0.912 (0.875–0.938)	91.3	88.9	89.3	90.1

At the optimal cutoff value in the validation set, the combined signature yielded a sensitivity of 91.3%, a specificity of 88.9%, a PPV of 89.3%, and a NPV of 90.1% (Table 8). Bootstrap-corrected AUC from the training process remained high (corrected AUC = 0.905), supporting the model's robustness.

### 3.5 Correlation between the four-miRNA signature and clinical phenotypes

To further substantiate the biological and clinical relevance of the four-miRNA signature, we investigated whether the diagnostic score, representing the integrated expression of the four miRNAs, correlated with the physiological indicators of HPG axis activation.

**Figure 5:**
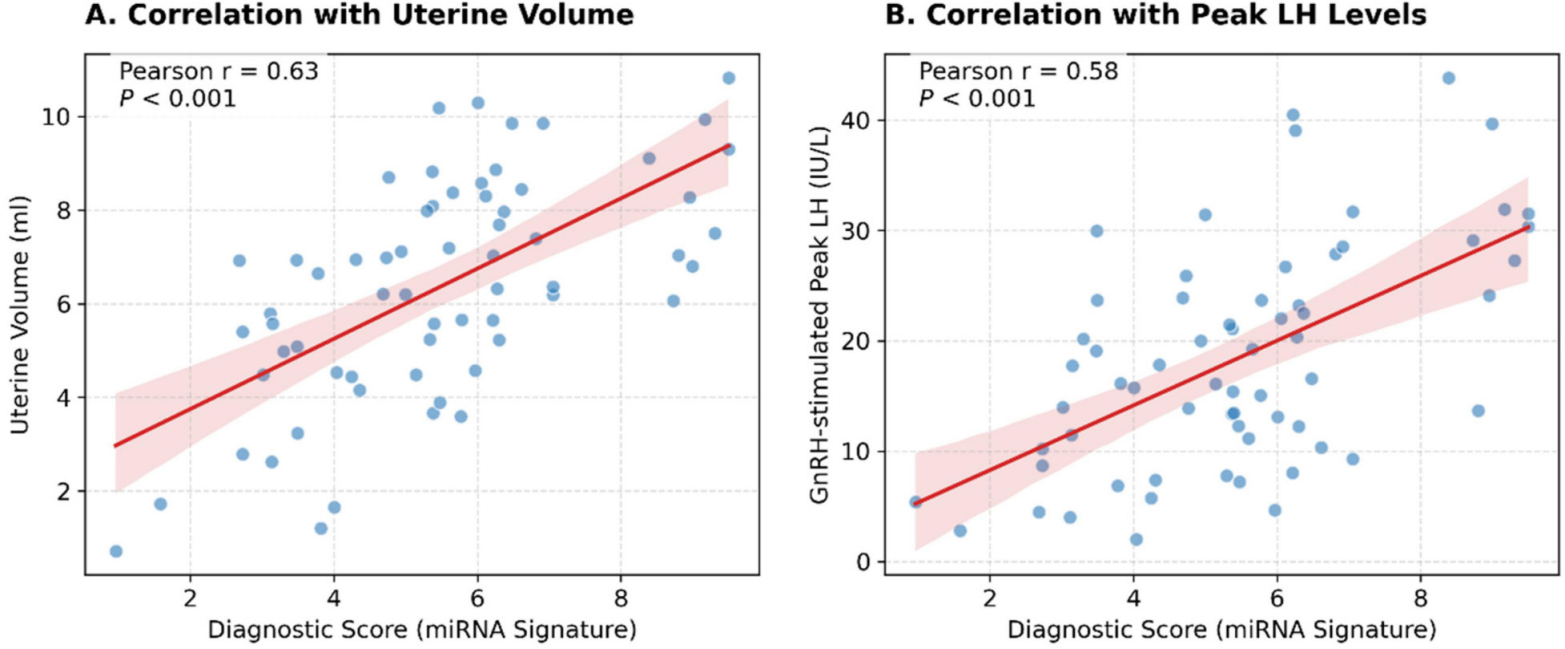
Correlation between the four-miRNA signature and clinical characteristics. Scatter plots showing the positive correlation between the integrated miRNA diagnostic score and **(A)** Uterine Volume (r = 0.63, *P* < 0.001) and **(B)** Peak LH levels (r = 0.58, *P* < 0.001). The linear regression line is shown in red.

Pearson correlation analysis revealed a significant positive correlation between the diagnostic score and uterine volume (r = 0.63, *P* < 0.001) in the CPP-naïve group (Figure 5A). This suggests that the aberrant expression of these miRNAs tracks with the end-organ response to estrogen exposure. Furthermore, the diagnostic score exhibited a positive correlation with peak LH levels (r = 0.58, *P* < 0.001) stimulated by GnRH (Figure 5B), indicating that the circulating exosomal miRNA profile quantitatively reflects the intensity of hypothalamic-pituitary activation. No significant correlation was observed with chronological age or BMI Z-score, reinforcing the specificity of the signature to pubertal development rather than general growth.

## 4 Discussion

The diagnosis of CPP in children remains a significant clinical challenge, primarily due to the reliance on the invasive, cumbersome, and often equivocal GnRH stimulation test (7, 9). This study addresses this critical gap by systematically profiling serum exosomal small RNAs to develop a non-invasive diagnostic tool. Our key findings are threefold: first, we identified a distinct serum exosomal small RNA signature that differentiates girls with treatment-naïve CPP from age-matched healthy controls; second, we developed and independently validated a robust four-miRNA diagnostic classifier with excellent performance (AUC = 0.912); and third, our bioinformatic analyses provide novel insights into the systems-level molecular dysregulation underlying CPP. These findings collectively establish a strong foundation for a non-invasive, molecularly-informed diagnostic paradigm for CPP.

This study is the first, to our knowledge, to systematically characterize the broader exosomal small RNA landscape in CPP using high-throughput sequencing. Our discovery phase revealed a widespread dysregulation not only of miRNAs but also of other small RNA species, including tsRNAs and piRNAs. This extends previous research that has largely focused on single RNA classes or candidate miRNAs (34). The distinct clustering of CPP and HC samples in both PCA and hierarchical clustering analyses strongly indicates that the exosomal miRNA profile reflects the underlying neuroendocrine state of pubertal activation. The identification of a stable, circulating molecular signature offers a powerful alternative to the fluctuating and functionally indirect nature of hormonal assays, paving the way for a more precise and patient-friendly diagnostic approach.

Beyond its diagnostic utility, our data offer valuable insights into the pathophysiology of CPP. While monogenic causes involving genes like MKRN3 and KISS1 explain a fraction of familial cases, the majority of idiopathic CPP cases remain mechanistically elusive (16, 17). Our systems-level approach provides a complementary perspective. The functional enrichment analysis of the 65 differentially expressed miRNAs revealed that their predicted target genes were most significantly enriched in the GnRH signaling pathway (FDR = 2.8 × 10^−^^1^⁰). This finding directly links the observed exosomal miRNA alterations to the core pathophysiological axis of CPP. Furthermore, the significant enrichment of the Insulin signaling and PI3K-Akt signaling pathways provides a potential molecular bridge between the well-documented epidemiological association of obesity and accelerated puberty and the onset of CPP (35, 36). These pathways are known to integrate metabolic signals with reproductive function, suggesting that the dysregulated miRNAs may mediate the impact of nutritional status on the HPG axis.

Our protein-protein interaction network analysis further solidified these connections by identifying key hub genes, KISS1, IGF1, ESR1, and LEPR, at the center of the regulatory network targeted by the dysregulated miRNAs. These genes are established “gatekeepers” of pubertal timing, controlling GnRH secretion, growth, and energy homeostasis (37–39). The fact that these critical nodes are regulated by a network of differentially expressed exosomal miRNAs suggests a complex, multi-layered post-transcriptional regulatory mechanism in CPP pathogenesis. This moves beyond a single-gene paradigm and highlights the importance of coordinated gene expression regulation in premature pubertal activation. The exosomal miRNAs identified in our study may originate from hypothalamic neurons or other relevant tissues and act as systemic signals reflecting or even contributing to this dysregulation.

From a clinical standpoint, our four-miRNA diagnostic signature demonstrates performance metrics that are competitive with, and in some aspects superior to, existing non-invasive or minimally invasive alternatives. While basal LH levels, anti-Müllerian hormone, and pelvic ultrasound are used to triage patients, they often suffer from limited sensitivity in early puberty, significant operator dependency, or lack of standardized cutoffs (40, 41). Our classifier achieved a high sensitivity (91.3%) and specificity (88.9%) in an independent validation cohort. The multivariate nature of the signature provides inherent robustness against the biological or technical variability of a single marker. Moreover, a qRT-PCR-based test is highly standardizable, scalable, and can be seamlessly integrated into routine clinical laboratory workflows, offering a practical and cost-effective solution for large-scale screening and diagnosis (42). Regarding clinical implementation, while ultracentrifugation was used for biomarker discovery in this study, the translation to routine practice can be facilitated by commercially available exosome precipitation kits and standard qRT-PCR workflows. Compared to the GnRH stimulation test, which requires multiple blood draws and specialist supervision, this serum-based miRNA test offers a simplified, minimally invasive, and scalable alternative.

A primary concern regarding exosome-based diagnostics is technical feasibility in a routine clinical setting. While ultracentrifugation was employed in this discovery phase to ensure high-purity isolation, this method is labor-intensive for daily practice. However, the translation of this four-miRNA signature to the clinic is increasingly viable. Currently, commercial exosome precipitation kits allow for rapid isolation from small serum volumes (< 1 mL) without expensive ultracentrifuges. Furthermore, qRT-PCR is a standard, cost-effective technology available in most hospital laboratories. Compared to the GnRH stimulation test, which requires potential hospitalization, multiple invasive blood draws over 90–120 minutes, and injection of stimulating agents, a serum exosomal miRNA test offers a “liquid biopsy” alternative. It captures the molecular state of the HPG axis from a single fasting blood sample, significantly improving patient compliance and reducing the psychological burden on pediatric patients.

Our study has several limitations that should be acknowledged. First, the sample size, while sufficient for a discovery and validation theoretical framework, requires expansion in large-scale, multi-center cohorts to confirm generalizability across different ethnicities. Second, regarding the mechanistic insights, we acknowledge that this study primarily focused on biomarker discovery and diagnostic model construction. While our bioinformatic analyses strongly implicated the GnRH and insulin signaling pathways, and identified KISS1 and IGF1 as hub targets, we did not perform *in vitro* functional validation experiments, such as dual-luciferase reporter assays or targeted knockdown/overexpression models in hypothalamic neuronal cells. Consequently, the direct regulatory relationship between the identified miRNAs and their predicted targets remains to be experimentally confirmed. This mechanistic validation is the dedicated focus of our team's ongoing follow-up study. Nevertheless, the strong correlation observed between the miRNA signature and clinical phenotypes (uterine volume and LH levels) provides indirect evidence of their functional involvement in CPP pathology.

## 5 Conclusion

In this study, we successfully identified and validated a novel, serum exosomal four-miRNA signature that accurately distinguishes girls with treatment-naïve CPP from healthy controls, achieving an AUC of 0.912 in an independent validation cohort. This molecular signature represents a significant advancement, offering a powerful, non-invasive alternative to the invasive GnRH stimulation test. Beyond clinical utility, our findings link circulating markers to the dysregulation of core pubertal networks, including the GnRH signaling pathway and key hub genes like *KISS1* and *IGF1*. Collectively, this work establishes a robust foundation for a clinically translatable diagnostic tool and advances the precision diagnosis of pediatric endocrinology.

## Data Availability

The original contributions presented in the study are publicly available. This data can be found here: https://ngdc.cncb.ac.cn/gsa-human/ (Accession number: HRA018349).
