# Supplementary material for: A serum exosomal four-miRNA signature for the diagnosis of central precocious puberty: a discovery and validation study
Source: Front Pediatr. 2026 May 14;13:1722017. doi: 10.3389/fped.2025.1722017 (PMC13218294; doi:10.3389/fped.2025.1722017)
Supplement: Supplementary file 1 [file Table1.docx]

**Supplementary Table 1. Quality metrics of small RNA sequencing**

| **Metric** | **Value** |
| --- | --- |
| Raw Reads per Sample (millions) | 12.1 ± 1.8 |
| High-Quality Reads per Sample (millions) | 11.2 ± 1.6 |
| Retention Rate (%) | 92.6 ± 2.1 |
| Q30 Score (%) | 96.3 ± 1.4 |
| Adapter Contamination (%) | < 1.2 |
